# Latent Profile Analysis of Depression and Its Influencing Factors Among Frail Older Adults in China

**DOI:** 10.3390/bs15091217

**Published:** 2025-09-08

**Authors:** Lingling Ye, Penghao Fan, Siyuan Zhang, Chao Rong

**Affiliations:** School of Humanities and Management, Zhejiang Chinese Medical University, Hangzhou 310053, China; 202421114611010@zcmu.edu.cn (L.Y.); 202321114611003@zcmu.edu.cn (P.F.); 202421114611012@zcmu.edu.cn (S.Z.)

**Keywords:** frailty, depression, mental health, older adults, latent profile analysis

## Abstract

The present investigation set out to examine potential categories regarding depressive symptoms in frail senior individuals in China and to identify the contributing variables associated with each category, with the goal of informing more targeted mental health interventions. Data were drawn from the 2018 China Health and Retirement Longitudinal Survey, commonly called CHARLS, which comprised an overall cohort of 1083 qualifying respondents. A latent profile analysis (LPA) revealed the following four distinct depression profiles: a Low Depression–High Loneliness Group (38.4%), a Moderately Low Depression–High Suicidal Ideation Group (7.5%), a Moderately High Depression–High Negative Emotion Group (33.4%), and a High Depression–High Suicidal Ideation Group (20.7%). Ordered multi-categorical logistic regression and restricted cubic spline analyses revealed that age, gender, body pain, pension insurance, sleep duration, and frailty index were significant predictors of depression classification. These findings suggest that depressive symptoms among frail older individuals in China are markedly heterogeneous, highlighting the need to develop differentiated intervention strategies for distinct depression risk groups to promote their mental health.

## 1. Introduction

At present, the growing number of older adults around the world has turned population aging into a serious global issue (Beard et al., 2016). China faces an even more serious challenge of aging due to its large population base. Data from the seventh national census indicate that China has 260 million individuals aged 60 and above, which corresponds with 18.70% of the overall population (Ning, 2021). It is predicted that by 2033, China will experience a stage of severe population aging, with over 20% of the population aged 60 and above, or at least 14% aged 65 and older, and this upward trend is anticipated to persist (Luo et al., 2021). Under this trend, the health problems of the aging population have become increasingly prominent, and the clinical syndrome of frailty is receiving increasing attention. Frailty, a widespread nonspecific condition in the older population, is typified by the deterioration of several physiological systems and heightened susceptibility to stressors (Fried et al., 2001; Hoogendijk et al., 2019; Morley et al., 2013). It can result in numerous negative health consequences, including depression, cognitive impairment, falls, fractures, and even death (Liu et al., 2022). Frailty has increasingly become a major public health concern impacting the wellbeing of older individuals (Hoogendijk et al., 2019). It is generally characterized by chronic and dynamic changes (Jang et al., 2021). Frailty prevalence rises significantly with age; individuals aged 75–84 exhibit a frailty prevalence of 15%, more than twice that of the 65–74 age group (6%), while those aged 85 and above reach 25%, over four times higher than the 65–74 cohort (Qiu et al., 2024; B. He et al., 2019). The frailty index (FI), an instrument for evaluating frailty among older adults, is calculated from the aggregated count of health deficits associated with aging (Hoogendijk et al., 2019). It is commonly applied to health-related research on older adults, and prior research has indicated that elevated FI scores correspond with a greater likelihood of unfavorable health consequences (Si et al., 2021). Therefore, accurately assessing the frailty status of older people and identifying related health concerns are important for optimizing health intervention strategies and delaying functional decline in this population.

In later life, depression is a common psychological disorder and is strongly associated with frailty (Kim et al., 2025). It is typically accompanied by functional impairment, increased utilization of healthcare services, and a decline in living standards (Bunce et al., 2012). It is a crucial measure for evaluating older people’s mental health. Moreover, recent studies have found that depression patients frequently lead unhealthy lives and have irregular eating habits, which predispose them to obesity and further compound their overall health burden (Ziółkowska et al., 2024). Globally, approximately 28.4% of older individuals experience depression (Hu et al., 2022). In China, depression affects older adults at a higher rate than adolescents, and depressed older adults are four to five times more likely to commit suicide than the general public (Rong et al., 2020). As population aging accelerates in China, depression among older individuals has become increasingly common and now constitutes a significant public health concern (Chen et al., 2023).

Scholarly attention has increasingly focused on the close linkage between frailty and mental health among older adults. Geriatric depression and frailty are both considered to be geriatric syndromes and are closely related to aging. There is a bidirectional association between these two conditions, and their interactions may stem from common pathophysiological mechanisms that contribute to their development and progression (Soysal et al., 2017). Some investigations have reported that frailty shows a strong connection with detrimental psychological consequences, such as depression, anxiety, and loneliness (Cagaloglu & Yılmaz, 2025; Kim et al., 2024; X. Wang et al., 2025). Older individuals identified as frail have approximately a fourfold greater likelihood of experiencing depression compared with individuals without frailty (Ní Mhaoláin et al., 2012). Escalating depressive symptoms among frail elders will further aggravate their frailty, thereby impairing their daily functioning and overall life satisfaction (Tang et al., 2023). In addition, the interaction between frailty and depression contributes to poorer outcomes in health, including elevated mortality, increased functional impairment, and a heightened risk of hospitalization (Zhao & Zhong, 2025). Although these studies have revealed the close relationship between frailty and depression, they tend to view frail older adults as one single category, ignoring the diversity of their internal depressive symptoms.

A latent profile analysis (LPA), a commonly used human-centered data analysis method, can identify underlying categorical differences among individuals based on observed variables. During the past few years, numerous investigations have employed LPAs to explore the latent characteristics pertaining to psychological wellbeing among senior citizens living under different circumstances or suffering from various diseases (Y. Wang & Zhang, 2024). However, there is limited research on LPAs for Chinese frail older adults, particularly with regard to identifying their potential categories of depression and their influencing factors. Therefore, this study utilizes the 2018 China Health and Retirement Longitudinal Study (CHARLS) dataset and employs an LPA in order to discern hidden patterns of depressive manifestations among frail Chinese seniors. Additionally, the present study examines potential influencing factors, including sociodemographic characteristics, physical health status, lifestyle, and social security, to optimize mental health intervention strategies for frail senior adults and promote these individuals’ overall wellbeing throughout later life.

## 2. Materials and Methods

### 2.1. Data Sources

This study made use of data from the China Health and Retirement Longitudinal Study (CHARLS), which is a nationally representative cross-disciplinary survey directed by the National School of Development at Peking University and implemented by the China Social Survey Center. The survey gathers comprehensive information on individuals’ demographic characteristics, family structure, health status, cognition and symptoms of depression, healthcare and public or private insurance, and employment, together with retirement status, etc. It seeks to collect reliable personal and household data from middle-aged and older adults in China, thereby offering a stronger scientific foundation to address aging-related challenges and inform policies for the older population. CHARLS encompasses 150 counties and 450 communities (including villages) across 28 provinces, autonomous regions, and municipalities in China, with a sample of around 19,000 respondents from 12,400 households. Ethical approval for the study was secured from the Institutional Review Board, and written informed consent was obtained from each participant prior to the interviews commencing.

### 2.2. Participants

**Figure 1:**
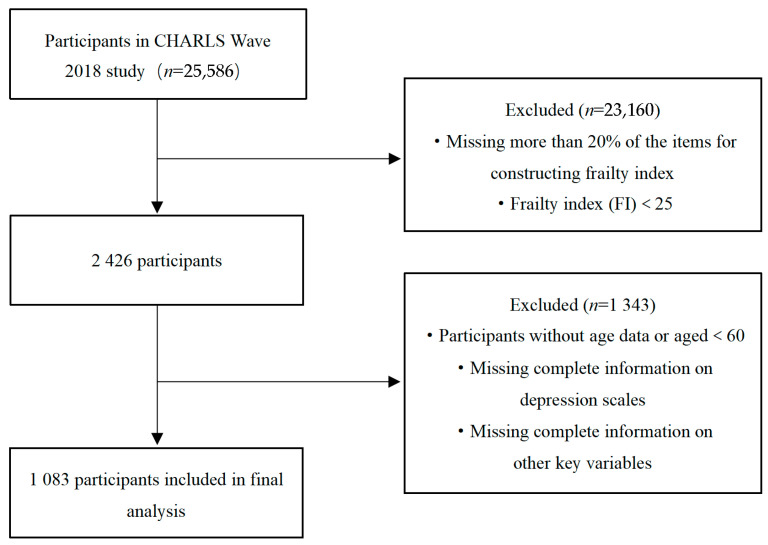
Flowchart of sample screening.

This study used CHARLS 2018 follow-up data and excluded the following participants: (1) age < 60 years; (2) incomplete depression scale information; (3) those missing more than 20% of the frailty index (FI) components; (4) an FI < 25; and (5) lack of information on other key variables. Finally, 1083 participants were incorporated into the study. Figure 1 illustrates the screening procedure.

### 2.3. Measures

#### 2.3.1. Study Variables

The sociodemographic characteristics involved in this study included gender, age, education level, type of residence, and marital status. Lifestyle indicators included smoking, drinking, and sleep duration. In terms of health status, the assessment included chronic disease prevalence and body pain experience. In addition, social security and social interactions were evaluated by participation in medical insurance, pension insurance, and social activities. Among them, the prevalence of chronic diseases was determined according to the self-reported information from the participants, and those who reported one or more chronic diseases were defined as suffering from chronic diseases. The specific variable assignment criteria are shown in Table 1.

#### 2.3.2. Frailty

The evaluation of participants’ frailty in the present research was conducted using the frailty index (FI). The FI is an indicator that serves to numerically gauge frailty severity and is derived by aggregating age-associated health shortfalls following well-accepted protocols reported in earlier research (D. He et al., 2023; Xie et al., 2025). Following the assessment of the 2018 CHARLS data, 32 items—covering comorbidities, physical function, disability, depression, and cognition—were selected to construct the FI (see Supplementary Materials Table S1). With the exception of item 32, the items were assigned as 0 or 1 according to the results of the respondents’ questionnaires, where 0 indicated no health deficit and 1 indicated a health deficit. Continuous scoring from 0 to 1 was applied to Item 32, where higher values represented poorer cognitive functioning (Searle et al., 2008). Respondents missing more than 20% of item responses were excluded from the scale analysis. The 32-FI was computed by adding up all observed deficits, dividing that figure by 32, then scaling the quotient by 100. The resulting 32-FI produced a continuous measure ranging from 0 to 100, where greater scores denoted more severe frailty. According to established precedents in the literature, frailty in this study was defined as a 32-FI ≥ 25 (Fan et al., 2020). The included subjects aged 60 and above met this criterion, so they were defined as frail older adults.

#### 2.3.3. Depression

In this study, the 10-item Center for Epidemiologic Studies Depression Scale, abbreviated as CESD-10, was used to evaluate participants’ depressive states (Andresen et al., 1994). The scale was measured by asking respondents about their feelings and behaviors during the preceding seven days, and comprised ten items, of which two were phrased positively and eight adopted negative wording. Responses for each item were scored as 0, 1, 2, or 3, aligning with “rarely or never (less than one day)”, “some of the time (one to two days)”, “occasionally or roughly half the time (three to four days)”, and “most of the week (five to seven days)”, with scoring reversed for positively phrased items. The overall score varied between 0 and 30, and a higher total denoted more severe depressive symptoms. The scale has been reported to possess sound reliability and construct validity, and serves as a suitable depression screening tool for older Chinese adults. The scale employed in the present investigation produced a Cronbach’s alpha of 0.766, signaling adequate internal consistency.

### 2.4. Statistical Analysis

In this study, Mplus (version 8.3) was adopted to examine the potential profiles of depression among Chinese frail older adults. The scores of each item in the CESD-10 questionnaire were used as exogenous variables, and 1~6 profiles were sequentially selected for the analysis. The LPA has four types of fitting indicators. (1) Model adequacy is judged using the Akaike Information Criterion (AIC), Bayesian Information Criterion (BIC), and adjusted Bayesian Information Criterion (aBIC), where lower scores signify a superior fit. (2) An entropy value approaching 1 suggests higher classification precision. (3) The Lo–Mendell–Rubin likelihood ratio (LMR) and the bootstrapped likelihood ratio test (BLRT) are utilized to compare models with *k* versus *k* – 1 classes. When the resulting *p*-value is below 0.05, the model with *k* classes is considered to yield a markedly improved fit relative to its *k* − 1 counterpart. (4) Each latent class comprises no less than five percent of the overall participants.

SPSS 25.0 and R 4.3.1 served as the primary tools for all statistical work. Non-normally distributed quantitative data were presented as [M(P25,P75)] and qualitative data as frequency (n) and percentage (%). In the univariate analysis, the nominal variables were examined using either the chi-squared test or Fisher’s exact test, whereas the ordinal variables and continuous variables that did not follow a normal distribution were assessed using the Kruskal–Wallis H test. An ordered multi-categorical logistic regression identified the factors influencing potential depression classifications in Chinese frail older adults. Dose–response relationships of the frailty index (FI) and sleep duration with depression categories were explored using a restricted cubic spline analysis, and the most suitable knot count was chosen based on the Akaike Information Criterion (AIC). A two-tailed significance threshold of an alpha value equal to 0.05 was adopted.

## 3. Results

### 3.1. Common Method Bias Test

As all variables in this study were gathered from participants’ self-reported questionnaires, Harman’s single-factor procedure was employed to mitigate potential common method bias, and the percentage of variance captured by the first factor served as the evaluation benchmark. The analysis revealed that this first factor accounted for only 24.97% of the overall variance, which is comfortably below the conventional 40% cutoff, suggesting that common method bias was not a serious issue in the present research and that the subsequent analyses could proceed without reservation.

### 3.2. Baseline Characteristics of Frail Older Adults

**Figure 2:**
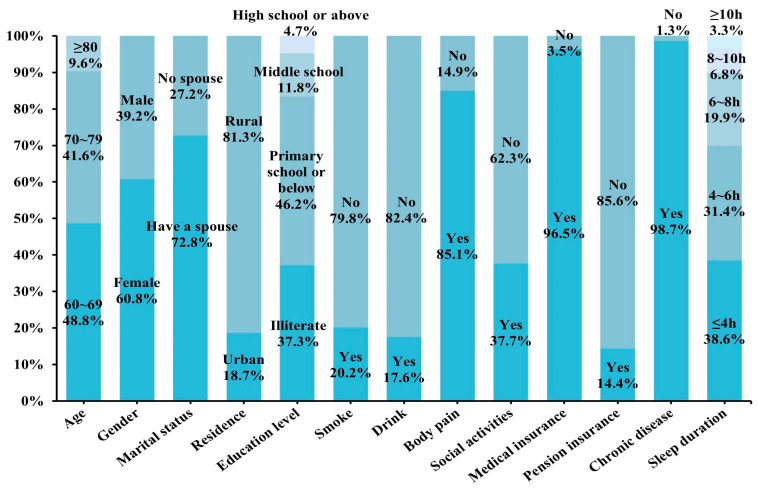
Demographic characteristics of Chinese frail older adults.

In this study, 60.8% of participants were female and 48.8% were aged between 60 and 69 years. A total of 72.8% of the older adults were married, 81.3% were from rural areas, and 46.2% had attained a primary school education or less. Of the older adults, 79.8% were non-smokers and 82.4% were non-drinkers; 85.1% had experienced body pain and 62.3% had not socialized in the last month. Nearly all older adults had medical insurance (96.5%), whereas only 14.4% possessed pension insurance. Additionally, 98.7% reported that they had chronic diseases, and 38.6% had fewer than 4 h of night-time sleep. Figure 2 presents the results in detail.

### 3.3. Latent Profiles of Depression in Frail Older Adults

#### 3.3.1. Latent Profile Model Fitting and Selection

Starting from the initial model, six latent profile models were fitted using depression assessment data from frail older adults in China. Table 2 presents the model fit statistics. The analysis indicated a gradual decrease in AIC, BIC, and aBIC values corresponding with the growing number of categories, reflecting a continuous improvement in the model fit, with Model 6 exhibiting the lowest values. However, the LMR test for Model 6 was not statistically significant (*p* > 0.05), so this model was excluded from consideration. In Model 5, one of the profiles accounted for only 3.3%, indicating that the classification was overly complex and lacked practical applicability. In contrast, Model 4 had the highest entropy value, and both the LMR and BLRT tests reached significance (*p* < 0.001), indicating that it provided the optimal model fit.

In addition, a discriminant analysis was used to assess the accuracy of the optimal model. Each potential category exhibited average posterior probabilities of 96.1–98.5%, indicating high classification accuracy. These results demonstrated good certainty and effective discrimination of the model classification, as detailed in Table 3.

#### 3.3.2. Classification and Features of Depressive Profiles in Frail Older Adults

**Figure 3:**
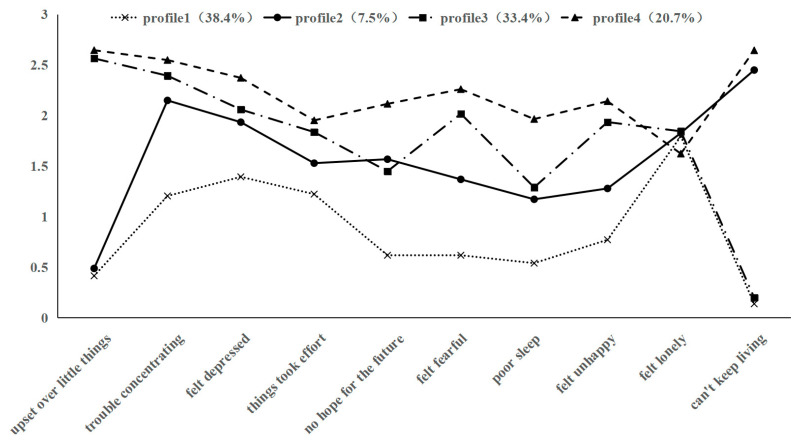
Distribution of characteristics across four latent depressive profiles in frail older adults.

Based on the latent profile analysis of Model 4, four groups of depressive symptom characteristics were identified and plotted (Figure 3). Profile 1 (38.4%) exhibited lower depression scores compared with other profiles, but scored relatively high on the “felt lonely” item. Therefore, it was labeled as the Low Depression–High Loneliness Group. Profile 2 (7.5%) had overall scores at a moderately low level, but showed a relatively high score on the “can’t keep living” item, so it was named the Moderately Low Depression–High Suicidal Ideation Group. Profile 3 (33.4%) scored at a moderately high level, with particularly prominent responses on negative emotion items such as “felt depressed”, “felt fearful”, and “felt unhappy”. Thus, it was named the Moderately High Depression–High Negative Emotion Group. Profile 4 (20.7%) demonstrated the highest depression scores among all profiles, particularly with elevated scores on the “can’t keep living” item, so we named it the High Depression–High Suicidal Ideation Group.

### 3.4. Univariate Analysis of Depression Profiles in Frail Older Adults

Table 4 indicates that age, gender, smoking, body pain, pension insurance, sleep duration, and FI were all significantly linked to the latent classes of depression among frail older individuals (*p* < 0.05).

### 3.5. Multivariate Analysis of Depression Profiles in Frail Older Adults

This study used the High Depression–High Suicidal Ideation Group as the reference group and treated the potential depression profiles of frail older adults as the dependent variable. Independent variables in the ordered multi-category logistic regression model were selected based on their statistical significance in the univariate analysis. The results of the parallel lines test were *χ*^2^ = 14.796 and *p* = 0.540, indicating the existence of the proportional dominance hypothesis. The results showed that age, gender, body pain, pension insurance, sleep duration, and FI had statistically significant effects (*p* < 0.05), suggesting that the heterogeneity of depression in frail older individuals might be explained by these factors (Table 5).

### 3.6. Dose–Response Relationship of FI and Sleep Duration with Latent Depressive Profiles in Frail Older Adults

**Figure 4:**
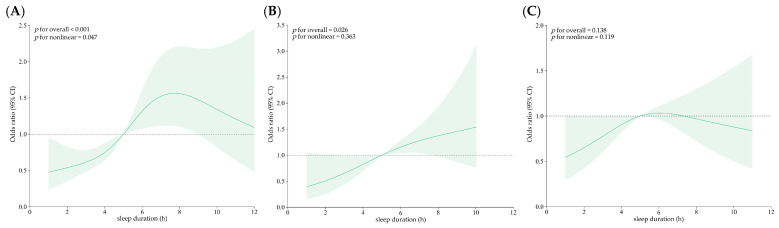
Relationship between sleep duration and latent profiles of depression in frail older adults. (**A**), (**B**), and (**C**) refer to the Low Depression–High Loneliness Group, Moderately Low Depression–High Suicidal Ideation Group, and Moderately High Depression–High Negative Emotion Group, respectively.

**Figure 5:**
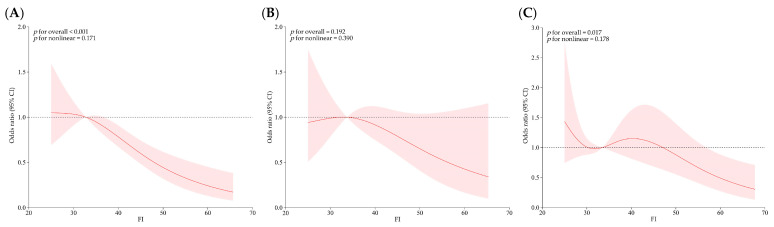
Relationship between the FI and latent profiles of depression in frail older adults. (**A**), (**B**), and (**C**) refer to the Low Depression–High Loneliness Group, Moderately Low Depression–High Suicidal Ideation Group, and Moderately High Depression–High Negative Emotion Group, respectively.

This study used the High Depression–High Suicidal Ideation Group as the reference group to analyze the effects of the FI and sleep duration on the other depression profiles, as shown in Figure 4 and Figure 5. In these figures, the horizontal axis depicts the continuous variations in sleep duration and FI, while the vertical axis represents the odds ratios (ORs) corresponding with each value of sleep duration and FI after controlling for confounding factors.

Sleep duration showed a nonlinear dose–response relationship with the risk of developing into the Low Depression–High Loneliness Group (*p* for overall < 0.001; *p* for nonlinear = 0.047). It was significantly related to the overall risk of entering the Moderately Low Depression–High Suicidal Ideation Group (*p* for overall = 0.026), but showed no significant nonlinear trend (*p* for nonlinear = 0.363). No significant association was observed for the Moderately High Depression–High Negative Emotion Group (*p* for overall = 0.138; *p* for nonlinear = 0.119).

Compared with the High Depression–High Suicidal Ideation Group, the FI was significantly associated with the overall risk of being in the Low Depression–High Loneliness Group (*p* for overall < 0.001) and Moderately High Depression–High Negative Emotion Group (*p* for overall = 0.017), but neither showed a significant nonlinear trend (*p* for nonlinear > 0.05). The FI was not significantly associated with the Moderately Low Depression–High Suicidal Ideation Group (*p* for overall = 0.192; *p* for nonlinear = 0.390).

## 4. Discussion

### 4.1. Analysis of Latent Depressive Profiles Among Frail Older Adults

Previous research has tended to examine the link between frailty and depression by simply adding up the points on a depression scale. This study utilized the LPA to identify four latent depressive profiles among frail older people in China. These findings clearly indicate significant heterogeneity in depressive symptoms among frail older people, refining the connection between frailty status and different psychological symptoms and facilitating more precise psychological interventions for the frail older population.

The results showed that 38.4% of frail older adults were classified into the Low Depression–High Loneliness Group. Individuals in this group did not exhibit obvious depressive symptoms, but commonly experienced a strong sense of loneliness. One reason is that frailty causes a decline in physical function among older adults, which restricts their ability to participate in social activities and interpersonal interactions, thereby affecting their social ties (Jin et al., 2020). Additionally, worsening health conditions could weaken frail older individuals’ ability to return support, which in turn adversely affects how they perceive and interpret supportive behaviors, rendering them more vulnerable to a sense of neglect (Von Dem Knesebeck & Siegrist, 2003) and further exacerbating their sense of loneliness. Notably, studies have shown that comorbid conditions, including anxiety, loneliness, and depression, can mutually exacerbate one another, accelerating psychological decline and leading to poorer treatment outcomes in older people (Igbokwe et al., 2020). Thus, children of frail older adults should increase their companionship time, communicate with them regularly, and give them patient attention. Community organizations should be encouraged to conduct regular visits to strengthen the connections between frail older people and their communities, thereby alleviating the social isolation caused by physical limitations.

The Moderately Low Depression–High Suicidal Ideation Group accounted for 7.5%. This group exhibited a high risk of suicidal tendencies. Although they may not have displayed intense negative emotions, they showed severe risks in terms of extreme cognitive patterns, making their condition highly concealed and difficult to detect. Relying solely on the total score from traditional depression screening tools may result in the omission of these high-risk individuals. A cohort study emphasized that identifying suicidal tendencies among frail older adults requires attention to emotional disorders contributing to their poor physical health as well as any history of suicide attempts (Almeida et al., 2016), rather than focusing solely on depression as a single triggering factor. For this type of frail older person, it is essential to strengthen the identification of suicide risk signals, establish a more refined psychological screening mechanism, and actively promote mental health care for older adults.

Of the frail older adults, 33.4% belonged to the Moderately High Depression–High Negative Emotion Group. These people had prominent negative emotions (such as depression, fear, and unhappiness) and showed clear symptoms of depression, but had not yet progressed to cognitive hopelessness or strong suicidal ideation. Some scholars have pointed out that due to the effects of aging, older adults experience greater difficulty in cognitive processing and regulation of emotions (Santorelli & Ready, 2015; Braver & West, 2011; Isaacowitz & Livingstone, 2014). Due to diminished executive functioning and limited social support, frail older individuals are inclined to rely on emotional repression and rumination to regulate negative emotions (Ji et al., 2022). However, these two methods are maladaptive forms of emotion regulation and are closely linked to the worsening and persistence of negative emotions (Lewis et al., 2015). Therefore, emotional management training for this population should be strengthened to help them develop more positive emotional coping strategies and reduce the accumulation of negative emotions.

Of the frail older adults, 20.7% were classified into the High Depression–High Suicidal Ideation Group. As the people in this group presented with severe depression accompanied by strong suicidal ideation, their mental health conditions require close attention. Previous research has indicated that perceived burdensomeness serves as a strong and stable predictor in the development of suicidal ideation (Bickford et al., 2021). Specifically, many frail older adults transition from being “providers” of financial support or physical care to “recipients” due to health-related issues, and this shift can often lead to a perceived sense of being a burden to others (Metze et al., 2015). It aggravates their doubts about the value of their own existence and makes them more likely to fall into despair. Therefore, early screening and psychological interventions for depression and suicide risk should be strengthened for this high-risk group and their sense of being needed and social connection should be enhanced through family support, peer communication, and social participation to reduce the risk of suicide.

### 4.2. Factors Influencing Latent Depressive Profiles Among Frail Older Adults

This study explored the factors affecting the different potential types of depression in frail older people through ordered multi-categorical logistic regression. The findings indicated that frail older adults aged 60–69 years were at a significantly higher risk of depression than those aged 80 years and above (OR = 1.839; *p* = 0.003). One possible explanation is that older adults aged 60~69 undergo a psychological transition from work to retirement, a shift often characterized by diminished social roles, decreased life satisfaction, and feelings of negative mood (Pinquart & Schindler, 2007; Dang et al., 2022). In addition, physical deterioration contributes to the inability to maintain their old lifestyles and social interactions. Elevated depressive symptoms may result from the dual impact of physiological decline and psychological adjustment. In contrast, frail seniors aged 80 and above experiencing and adapting to changes in social roles have a higher level of acceptance of declining physical functioning and reduced social participation, resulting in relatively lower levels of depression.

In terms of gender differences, the level of depression in frail older women was 1.454 times higher than that in men (OR = 1.454; *p* = 0.005). Functional impairments have been reported to affect depressive symptoms more strongly in women (Guo et al., 2024). Women are more likely to have negative emotions in the face of the gradual deterioration of physical function, which in turn induces or aggravates depressive symptoms. The manners in which men and women cope with negative emotions are different. Men tend to cope with stress through substance use, such as smoking and drinking, whereas women primarily rely on emotional inhibition (Nolen-Hoeksema, 2012). This approach is not effective in relieving psychological stress and may lead to emotional dysfunction over time, significantly increasing the risk of depression.

As an important physiological factor, body pain also significantly impacts the degree of depression in frail older people (OR = 1.474; *p* = 0.021). Individuals who suffer from body pain typically experience impaired physical functioning and may have accompanying mental health concerns, which can lead to diminished life quality and significantly impair overall health (Cohen et al., 2021). Within the current investigation, physically vulnerable older people generally had body pain problems. Persistent pain stimulation can result in decreased physical movement in older adults, restricting their capacity to take part in routine tasks and social interactions, thereby increasing feelings of social isolation and raising the likelihood of depression.

The findings indicated that taking part in pension insurance corresponded with a markedly lower likelihood of depression in frail seniors (OR = 0.676; *p* = 0.020). Compared with the financially insecure, frail older adults who receive pension insurance have a notably reduced chance of depression. Frail older people often suffer from chronic illnesses, which not only adds to the burden of healthcare costs, but also tends to trigger anxiety and depression. In this context, social protection mechanisms play a crucial role. Public welfare and pension insurance can ease life pressures for older people, enhance their recognition of the fairness of income redistribution, and help to promote mental health (Chai et al., 2022).

Sleep duration serves as an important protective factor for depression among frail older adults (OR = 0.914 *p* < 0.001), while insufficient sleep significantly increases the risk of developing depression. The RCS revealed that different potential classes of depression exhibit varying sensitivities to changes in sleep duration. Sleep duration had a nonlinear dose–response association with the risk of being in the Low Depression–High Loneliness Group. A moderate sleep duration of 6–8 h provides the strongest protective effect against depression risk, while both insufficient sleep (fewer than 5 h) and excessive sleep (more than 8 h) significantly increase the likelihood of developing depression. Some studies have found that keeping a regular and healthy sleep routine may mitigate the risk of frailty or pre-frailty status (Zhu et al., 2022). An abnormal sleep duration, whether too long or too short, is closely associated with frailty status (Balomenos et al., 2021; Moreno-Tamayo et al., 2021). Consequently, maintaining a moderate sleep duration not only helps to delay the decline in physical function, but also effectively reduces the risk of depression.

The FI serves as a quantitative measure of health deficits in older people and positively correlates with depression severity (OR = 1.028; *p* < 0.001). The RCS revealed that the FI exhibited a significant relationship with Low Depression–High Loneliness Group and Moderately High Depression–High Negative Emotion Group. This suggested that increased frailty may limit older individuals’ social engagement, increase feelings of loneliness, and lead to more negative emotions like sadness and unhappiness, which can ultimately worsen depression. No notable link was found between the FI and Moderately Low Depression–High Suicidal Ideation Group, suggesting that for older adults with strong suicidal ideation, the mechanisms underlying depression may be more complex and less influenced by physical decline.

## 5. Limitations

The present study is limited in certain aspects. Firstly, its cross-sectional nature does not allow for causal inferences regarding the association between potential influencing factors and depression subtypes in frail seniors. Secondly, the research data is primarily derived from self-reported responses provided by respondents in the 2018 CHARLS. These reports may be subject to memory and social expectation biases, particularly among older adults. Measurement tools such as the CESD-10 scale and FI are influenced by subjective factors and construction methods, which may lead to certain biases in the assessment results of depressive symptoms and frailty. Thirdly, as with other model-based clustering methods, the results of the LPA may be influenced by the researcher’s selection of the optimal number of classes and by potential measurement errors in the observed variables, which could affect the robustness and reproducibility of the classification. Finally, the scope of this study is limited to China, and other regions may have different results due to living habits and cultural differences. In future studies, a longitudinal design could be adopted and the sample area expanded to continuously track changes in frailty and depression, thereby providing a basis for developing more effective intervention measures.

## 6. Conclusions

In summary, this study applied an LPA to categorize Chinese frail older people into the following four potential depression profiles: a Low Depression–High Loneliness Group, a Moderately Low Depression–High Suicidal Ideation Group, a Moderately High Depression–High Negative Emotion Group, and a High Depression–High Suicidal Ideation Group. The findings revealed that factors such as gender, age, physical pain, pension insurance, sleep duration, and frailty index played a key role in determining depression categories among frail older people, demonstrating that depressive manifestations in this population are highly heterogeneous. Based on the depressive characteristics of different potential categories, tailored intervention strategies should be developed, such as strengthening emotional support to alleviate loneliness, establishing suicide crisis intervention mechanisms, and guiding positive emotional regulation. Furthermore, leveraging community resources to promote precise interventions and strengthen social support is essential for improving the psychological health of frail older adults. Beyond targeted screening and psychosocial support, growing evidence indicates that non-pharmacological interventions—such as physical exercise, social engagement activities, and acupressure—can help to reduce psychological burdens in older people (Tao et al., 2023; Lin et al., 2022). Therefore, adopting a multidimensional approach that integrates these strategies is essential to safeguard the psychological health and overall life satisfaction of frail older adults. At the policy level, the psychological issues of the frail older population should be incorporated into the national mental health service system. Efforts should be made to further normalize depression risk screening and strengthen collaboration between sectors in mental health services. Such measures will help to keep frail older adults healthy in body and mind at the institutional level, ultimately enhancing their quality of life.

## Figures and Tables

**Table 1:** Assignment of independent variables.

Variable	Assignment Mode
Age	1 = “60–69”; 2 = “70–79”; 3 = “≥80”
Gender	1 = “Female”; 2 = “Male”
Marital status	1 = “Have a spouse”; 2 = “No spouse”
Residence	1 = “Urban”; 2 = “Rural”
Education level	1 = “Illiterate”; 2 = “Primary school or below”
	3 = “Middle school”; 4 = “High school or above”
Smoke	1 = “Yes”; 2 = “No”
Drink	1 = “Yes”; 2 = “No”
Body pain	1 = “Yes”; 2 = “No”
Social activities	1 = “Yes”; 2 = “No”
Medical insurance	1 = “Yes”; 2 = “No”
Pension insurance	1 = “Yes”; 2 = “No”
Chronic disease	1 = “Yes”; 2 = “No”

**Table 2:** Comparison of LPA model-fitting index.

Model	K	Likelihood	AIC	BIC	aBIC	Entropy	LMR	BLRT	Categorical Probability
1	20	−17,568.224	35,176.449	35,276.198	35,212.674	—	—	—	—
2	31	−16,634.041	33,330.083	33,484.695	33,386.233	0.891	0.0000	0.0000	0.452/0.548
3	42	−16,221.137	32,526.275	32,735.749	32,602.348	0.906	0.0000	0.0000	0.281/0.340/0.380
4	53	−16,025.617	32,157.233	32,421.570	32,253.231	0.956	0.0000	0.0000	0.075/0.207/0.334/0.384
5	64	−15,894.023	31,916.047	32,235.246	32,031.969	0.934	0.0000	0.0000	0.033/0.176/0.247/0.247/0.296
6	75	−15,757.079	31,664.157	32,038.219	31,800.003	0.930	0.1214	0.0000	0.077/0.095/0.152/0.202/0.219/0.255

**Table 3:** Attribution probabilities for each latent profile across classes.

Class	Profile 1	Profile 2	Profile 3	Profile 4
1	0.978	0.000	0.022	0.000
2	0.000	0.961	0.000	0.039
3	0.022	0.001	0.977	0.000
4	0.000	0.015	0.000	0.985

**Table 4:** Univariate analysis of latent profiles of depression in frail older adults.

Variable	Low Depression–High Loneliness Group, n = 416 (38.4%)	Moderately Low Depression–High Suicidal Ideation Group, n = 81 (7.5%)	Moderately High Depression–High Negative Emotion Group, n = 362 (33.4%)	High Depression–High Suicidal Ideation Group, n = 224 (20.7%)	*χ* ^2^ */H*	*p*
Age					11.505	0.009
60~69	184 (44.23%)	36 (44.44%)	183 (50.55%)	125 (55.80%)		
70~79	176 (42.31%)	42 (51.85%)	149 (41.16%)	84 (37.50%)		
≥80	56 (13.46%)	3 (3.70%)	30 (8.29%)	15 (6.70%)		
Gender					25.932	<0.001
Female	223 (53.61%)	54 (66.67%)	216 (59.67%)	165 (73.66%)		
Male	193 (46.39%)	27 (33.33%)	146 (40.33%)	59 (26.34%)		
Marital status					3.841	0.279
Have a spouse	304 (73.08%)	60 (74.07%)	272 (75.14%)	152 (67.86%)		
No spouse	112 (26.92%)	21 (25.93%)	90 (24.86%)	72 (32.14%)		
Residence					2.656	0.448
Urban	85 (20.43%)	15 (18.52%)	68 (18.78%)	34 (15.18%)		
Rural	331 (79.57%)	66 (81.48%)	294 (81.22%)	190 (84.82%)		
Education level					5.828	0.120
Illiterate	144 (34.62%)	32 (39.51%)	132 (36.46%)	96 (42.86%)		
Primary school or below	196 (47.12%)	33 (40.74%)	170 (46.96%)	101 (45.09%)		
Middle school	59 (14.18%)	11 (13.58%)	40 (11.05%)	18 (8.04%)		
High school or above	17 (4.09%)	5 (6.17%)	20 (5.52%)	9 (4.02%)		
Smoke					8.312	0.040
Yes	86 (20.67%)	12 (14.81%)	87 (24.03%)	34 (15.18%)		
No	330 (79.33%)	69 (85.19%)	275 (75.97%)	190 (84.82%)		
Drink					5.594	0.133
Yes	74 (17.79%)	12 (14.81%)	75 (20.72%)	30 (13.39%)		
No	342 (82.21%)	69 (85.19%)	287 (79.28%)	194 (86.61%)		
Body pain					15.824	<0.001
Yes	335 (80.53%)	68 (83.95%)	313 (86.46%)	206 (91.96%)		
No	81 (19.47%)	13 (16.05%)	49 (13.54%)	18 (8.04%)		
Social activities					2.305	0.512
Yes	161 (38.70%)	35 (43.21%)	135 (37.29%)	77 (34.38%)		
No	225 (61.30%)	46 (56.79%)	227 (62.71%)	147 (65.63%)		
Medical insurance					7.526	0.057
Yes	396 (95.19%)	78 (96.30%)	357 (98.62%)	214 (95.54%)		
No	20 (4.81%)	3 (3.70%)	5 (1.38%)	10 (4.46%)		
Pension insurance					13.324	0.006
Yes	77 (18.51%)	11 (13.58%)	49 (13.54%)	19 (8.48%)		
No	339 (81.49%)	70 (86.42%)	313 (86.46%)	205 (91.52%)		
Chronic disease					2.317	0.470
Yes	411 (98.80%)	80 (98.77%)	355 (98.07%)	223 (99.55%)		
No	5 (1.20%)	1 (1.23%)	7 (1.93%)	1 (0.45%)		
Sleep duration	6.00 (4.00, 7.00)	5.50 (4.00, 7.25)	5.00 (3.38, 7.00)	4.25 (3.00, 6.00)	28.742	<0.001
Frailty index	31.25 (27.59, 37.50)	33.48 (28.13, 39.90)	33.15 (27.65, 39.76)	33.98 (28.13, 43.72)	12.550	0.006

Note: Values for sleep duration and frailty index are presented as the median and interquartile range (P25 and P75).

**Table 5:** Ordered multi-categorical logistic regression analysis of factors of latent depressive profiles in frail older adults.

Variable	*β*	Wald *χ*^2^	*OR*	95% *CI*	*p*
Low Depression–High Loneliness Group	0.977	6.951	2.656	1.285~5.490	0.008
Moderately Low Depression–High Suicidal Ideation Group	1.302	12.293	3.677	1.775~7.614	<0.001
Moderately High Depression–High Negative Emotion Group	2.899	58.226	18.156	8.628~38.245	<0.001
Age					
60~69	0.609	8.605	1.839	1.224~2.759	0.003
70~79	0.365	3.036	1.441	0.955~2.173	0.081
≥80	—	—	—	—	—
Gender					
Female	0.374	7.926	1.454	1.121~1.885	0.005
Male	—	—	—	—	—
Smoke					
Yes	0.082	0.281	1.085	0.801~1.473	0.596
No	—	—	—	—	—
Body pain					
Yes	0.388	5.314	1.474	1.060~2.048	0.021
No	—	—	—	—	—
Pension insurance					
Yes	−0.392	5.417	0.676	0.486~−0.940	0.020
No	—	—	—	—	—
Sleep duration	−0.090	14.885	0.914	0.874~−0.957	<0.001
Frailty index	0.028	22.707	1.028	1.017~1.041	<0.001

## Data Availability

This study utilized publicly accessible datasets, which are available at https://charls.pku.edu.cn/gy/gyxm.htm (accessed on 1 April 2025).

## Supplementary Materials

The following supporting information can be downloaded at https://www.mdpi.com/article/10.3390/bs15091217/s1, Table S1: The 32 items used to construct the frailty index.
