# Supplementary material for: Latent Profile Analysis of Depression and Its Influencing Factors Among Frail Older Adults in China
Source: Behav Sci (Basel). 2025 Sep 8;15(9):1217. doi: 10.3390/bs15091217 (PMC12466371; doi:10.3390/bs15091217)
Supplement: Supplementary file 1 [file behavsci-15-01217-s001.zip › behavsci-3747371-supplementary.pdf]

**Table S1** The 32 items used to construct the frailty index

| Items | Description of the items | Cut-off value |
|---|---|---|
| Self-Reported Diseases | 1.Physician diagnosed hypertension | Yes = 1, No = 0 |
| | 2.Physician diagnosed diabetes | Yes = 1, No = 0 |
| | 3.Physician diagnosed heart disease | Yes = 1, No = 0 |
| | 4.Physician diagnosed stroke | Yes = 1, No = 0 |
| | 5.Physician diagnosed cancer | Yes = 1, No = 0 |
| | 6.Physician diagnosed arthritis | Yes = 1, No = 0 |
| | 7.Physician diagnosed chronic lung disease | Yes = 1, No = 0 |
| | 8.Physician diagnosed asthma | Yes = 1, No = 0 |
| | 9.Physician diagnosed any emotional, nervous, or psychiatric problems | Yes = 1, No = 0 |
| | 10.Physician diagnosed memory-related disease | Yes = 1, No = 0 |
| Self-Reported Vision and Hearing Problems | 11.Self-reported eyesight (while using lenses if appropriate) | Yes = 1, No = 0 |
| | 12.Self-reported hearing (while using hearing aid if appropriate) | Yes = 1, No = 0 |
| Self-Reported General and Mental Health | 13.Self-reported general health status | Poor or fair = 1, excellent, very good, or good = 0 |
| | 14.Depression: CESD-10 questionnaire | CESD-10 >10 =1, ≤10 =0 |
| Activities of Daily Living (ADLs) | 15.Difficulty with dressing | Yes = 1, No = 0 |
| | 16.Difficulty with bathing or showering | Yes = 1, No = 0 |
| | 17.Difficulty with eating | Yes = 1, No = 0 |
| | 18.Difficulty with getting in and out of bed | Yes = 1, No = 0 |
| | 19.Difficulty with using the toilet | Yes = 1, No = 0 |
| Instrumental Activities of Daily Living (IADLs) | 20.Difficulty with managing money | Yes = 1, No = 0 |
| | 21.Difficulty with taking medications | Yes = 1, No = 0 |
| | 22.Difficulty with shopping for groceries | Yes = 1, No = 0 |
| | 23.Difficulty with preparing meals | Yes = 1, No = 0 |
| | 24.Difficulty with doing housework | Yes = 1, No = 0 |
| Mobility Status | 25.Difficulty with walking 100 yards | Yes = 1, No = 0 |
| | 26.Difficulty with getting up from a chair after sitting for long periods | Yes = 1, No = 0 |
| | 27.Difficulty with climbing several flights of stairs without resting | Yes = 1, No = 0 |
| | 28.Difficulty with lifting or carrying weights over 10 pounds/jins | Yes = 1, No = 0 |
| | 29.Difficulty with picking up a coin from the table | Yes = 1, No = 0 |
| | 30.Difficulty with stooping, kneeling, or crouching | Yes = 1, No = 0 |
| | 31.Difficulty with reaching arms above shoulder level | Yes = 1, No = 0 |
| Cognition Status | 32.(memory test score + orientation test score) / 14 | Continuous, ranging from 0 to 1 |

Heart disease indicates the angina, coronary heart disease, congestive heart failure, or other heart problems.

Memory-related disease indicates Alzheimer's disease or dementia, organic brain senility, or other serious memory impairment.

Depression is evaluated using Center for Epidemiologic Studies Depression Scale (CESD). In the CHARLS, CESD-10 is used, and the total score ranges from 0 to 30.

The memory score is the average of words that are not recalled in the immediate and delayed word recall tasks. The memory score ranges from 0 to 10. The orientation test comprises four questions about the day of the week, the month, the date of the month, and the year. One point is given for each wrong answer, and the range is from 0 to 4.
